# Radiographic Changes and Recorded Complications After Nonabsorbable Endosinotarsal Screw Arthroereisis for Symptomatic Pediatric Flexible Flatfoot: A Retrospective Cohort Study

**DOI:** 10.3390/jcm15207950

**Published:** 2026-10-14

**Authors:** Sergio De Salvatore, Costanzo Testa, Silvia Salera, Leonardo Oggiano, Edoardo Costici, Fabrizio Donati, Pier Luigi Maglione, Paolo Brigato, Rosa Russo, Pier Francesco Costici

**Affiliations:** 1 Orthopaedics and Traumatology Unit, Department of Surgery, Bambino Gesù Children’s Hospital, 00165 Rome, Italy; 2 Department of Orthopaedics and Traumatology, Fondazione Policlinico Universitario Agostino Gemelli IRCCS, 00136 Rome, Italy; 3 Department of Orthopaedics and Traumatology, Policlinico Tor Vergata, 00133 Rome, Italy; 4 Faculty of Medicine and Surgery, Saint Camillus International University of Health Sciences, 00131 Rome, Italy; 5 Research Unit of Orthopaedic and Trauma Surgery, Department of Medicine and Surgery, Università Campus Bio-Medico di Roma, Via Alvaro del Portillo 21, 00128 Rome, Italy

**Keywords:** flexible flatfoot, pes planovalgus, subtalar arthroereisis, endosinotarsal screw, pediatric orthopedics, radiographic outcomes

## Abstract

**Background**: Subtalar arthroereisis is used for selected children with symptomatic flexible flatfoot that persists despite nonoperative management, but published evidence is limited by heterogeneous indications and frequent failure to account for bilateral observations. This study evaluated radiographic change and the complications recorded after nonabsorbable endosinotarsal screw arthroereisis. **Methods**: We retrospectively reviewed consecutive patients aged 8–15 years treated at a tertiary pediatric orthopedic center between 2018 and 2024. Eligible patients had symptomatic flexible planovalgus deformity, failure of at least 6 months of conservative treatment, isolated screw arthroereisis, and complete weight-bearing radiographs obtained preoperatively and at 12 months postoperatively. Meary’s angle, the Costa–Bertani angle, Kite’s angle, and calcaneal pitch were analyzed using linear mixed-effects models with repeated observations at the foot level nested within patients. **Results**: Seventy-four patients (141 feet) were included in the outcome analysis; 67 patients (90.5%) underwent bilateral procedures. Mean age was 11.5 ± 1.6 years, and radiographic outcomes were assessed at 12 months in all patients; mean clinical follow-up was 18.3 ± 3.7 months. Interobserver reliability was good to excellent. Meary’s angle decreased from 19.0° ± 8.5° to 7.7° ± 5.6° (*p* < 0.001). The Costa–Bertani angle decreased by 9.0° (*p* < 0.001), Kite’s angle by 5.5° (*p* < 0.001), and calcaneal pitch increased by 1.7° (95% CI, 1.0 to 2.4; Holm-adjusted *p* = 0.001). Sinus tarsi pain occurred in 13 patients (17.6%) and resolved conservatively in 11. Implant removal was required in two patients (2.7%), and one patient underwent reoperation for loss of correction (1.4%). No deep infection or neurovascular injury was recorded. **Conclusions**: Nonabsorbable endosinotarsal screw arthroereisis was associated with radiographic improvement at the standardized 12-month assessment. Major recorded adverse events and reoperations were uncommon among the patients with available follow-up, whereas residual sinus tarsi pain was recorded in approximately one in six patients, and the implant was not removed in 97.3% of patients. The postoperative mean values indicate partial correction rather than uniform radiographic normalization. Because patient-reported and functional outcomes were unavailable, clinical benefit cannot be inferred from angular changes alone.

## 1. Introduction

Flexible flatfoot is characterized by collapse of the medial longitudinal arch during weight bearing, hindfoot valgus, and variable forefoot abduction, with reconstitution of the arch when the foot is unloaded or during heel rise. It is common in childhood, and prevalence estimates vary markedly with age, population, and diagnostic method [1,2,3,4]. Most flexible flatfeet are physiologic and asymptomatic; observation and reassurance are therefore appropriate in the absence of pain or functional limitation [1,2,5]. Treatment is directed toward the smaller subgroup with persistent symptoms, activity limitation, shoe-wear difficulty, or associated Achilles–gastrocnemius tightness [1,6].

Initial management usually consists of education, activity modification, stretching when equinus is present, and a trial of orthoses or physiotherapy. Evidence that orthoses alter the natural history of an asymptomatic flexible flatfoot remains limited, although they may improve symptoms in selected children [6]. Surgery should consequently be reserved for persistent symptomatic deformity after an adequate nonoperative trial and after exclusion of rigid, neurologic, syndromic, and coalition-related causes [1,2].

Subtalar arthroereisis limits excessive subtalar eversion by placing an implant within the sinus tarsi or tarsal canal. The procedure is attractive because it is minimally invasive and motion preserving, but implant design, operative technique, indications, and outcome measures vary substantially among published series [7,8,9,10,11,12,13,14,15,16,17]. Reported adverse events include sinus tarsi pain, under- or overcorrection, implant extrusion, and unplanned removal [7,9,13,15,16]. Bioabsorbable implants avoid permanent hardware but introduce material-specific considerations; current evidence does not establish definitive superiority of either absorbable or nonabsorbable devices [10,11,18].

Interpretation of pediatric arthroereisis studies is also complicated by the imperfect relationship between static morphology and symptoms. Symptomatic and asymptomatic flexible flatfeet may show considerable overlap in conventional radiographic measures, indicating that pain and functional limitation cannot be inferred from the degree of valgus or arch collapse alone [5]. Conversely, a reduction in excessive pronatory mechanics may still be relevant even when postoperative angles do not reach a predefined normal range. Studies that combine imaging with gait analysis, return to sport, or patient-reported outcomes are therefore particularly informative [19,20,21]. Bilateral surgery creates an additional analytical challenge because treating both feet as independent observations underestimates uncertainty. The purpose of this study was to quantify radiographic change and to describe the complications recorded after isolated nonabsorbable endosinotarsal screw arthroereisis for symptomatic flexible flatfoot, using mixed-effects models to account for repeated measurements and bilateral clustering. We hypothesized that all four radiographic parameters would improve after surgery and that major implant-related complications would be uncommon.

## 2. Materials and Methods

### 2.1. Study Design and Setting

This retrospective cohort study included consecutive patients treated at a single tertiary pediatric orthopedic center in Rome, Italy, between January 2018 and December 2024. The study was conducted in accordance with the ethical standards for clinical research set out in the Declaration of Helsinki. The institutional authority responsible for the review of clinical research at the treating centre is the Comitato Etico of the Ospedale Pediatrico Bambino Gesù, Rome, Italy (Ethics Committee of Bambino Gesù Children’s Hospital). Under the regulations of that committee, formal ethics committee approval was not required for this retrospective analysis because all data had been collected during routine clinical care and were fully anonymized before analysis. For the same reasons, the requirement for individual informed consent was waived. No separate determination document or protocol number was issued, because the local institutional review board regulations exempt retrospective studies of this kind from formal review. Under the policies of our institution, and in accordance with Regulation (EU) 2016/679 (General Data Protection Regulation), Italian Legislative Decree No. 101/2018, and the guidance issued by the Italian Data Protection Authority, neither ethics committee approval nor individual informed consent is mandatory for retrospective analyses of fully anonymized data collected as part of standard clinical practice. The study is reported according to the Strengthening the Reporting of Observational Studies in Epidemiology (STROBE) statement. Particular attention was given to the STROBE items concerning missing data, participant attrition, potential sources of bias, and the explicit distinction between the eligible, radiographic, and safety populations. A completed STROBE checklist is provided as Supplementary Material.

### 2.2. Participants

Eligibility criteria were age 8–15 years at surgery; symptomatic flexible planovalgus deformity with collapse of the medial longitudinal arch, hindfoot valgus, and forefoot abduction; restoration of the arch on heel-rise and Jack tests; failure of at least 6 months of conservative management with custom-molded orthoses and structured physical therapy; isolated nonabsorbable endosinotarsal screw implantation without concomitant osseous procedures; and complete standing anteroposterior and lateral radiographs obtained within 3 months before surgery and at 12 months after surgery. Denis grade III morphology was determined from the plantar footprint and was used as a severity descriptor rather than as an isolated indication for surgery.

#### 2.2.1. Definition of Symptomatic Deformity and Operative Indication

Deformity was considered symptomatic when the child reported, despite adequate nonoperative treatment, at least one of the following: activity-related pain in the medial arch, sinus tarsi, or posteromedial ankle region; fatigue or pain on walking or prolonged standing that limited daily or school activity; reduced participation in sport; or footwear intolerance attributable to hindfoot valgus. The most frequent presenting complaint was activity-related pain, followed by fatigue on walking or prolonged standing, and the pain was located principally in the medial arch. By definition of the eligibility criteria, symptoms persisted throughout the period of at least 6 months of nonoperative treatment that preceded the decision to operate. In the large majority of bilaterally treated children both feet were symptomatic and met the criteria above independently. In a minority, whose number was not recorded separately in the clinical database, the dominant symptoms were mainly unilateral and the contralateral foot was treated during the same session because of a comparable planovalgus deformity with only minor symptoms, to avoid a second anesthetic episode. This indication is acknowledged as a source of heterogeneity within the cohort.

All children underwent a standardized clinical examination performed by the operating surgeons. This comprised assessment of medial arch reconstitution on the heel-rise and Jack tests, evaluation of gastrocnemius-soleus tightness with the Silfverskiöld test, palpation of the sinus tarsi and of the posterior tibial tendon to identify alternative pain generators, and assessment of hindfoot alignment in stance. Generalized ligamentous laxity was assessed clinically, but the Beighton score was not recorded consistently in the clinical notes and was therefore unavailable as a study variable. Rigid deformity, tarsal coalition, and congenital vertical talus were excluded clinically and radiographically. The operative indication was agreed jointly by the two treating surgeons within the institutional treatment pathway for pediatric flexible flatfoot.

Nonoperative treatment before surgery consisted of custom-molded foot orthoses worn in regular footwear during weight-bearing activity, combined with a supervised physiotherapy program. The program included gastrocnemius-soleus and plantar fascia stretching when the Silfverskiöld test was positive, progressive strengthening of the tibialis posterior and of the intrinsic foot musculature, and proprioceptive and gait retraining, delivered in two supervised sessions per week for a minimum of 8 weeks and continued thereafter as a home program. Orthoses were worn for at least 6 months. Adherence to orthotic wear and to physiotherapy was recorded only qualitatively in the clinical notes and was not measured with a standardized instrument. The nonoperative program was therefore not necessarily identical in every child.

#### 2.2.2. Study Populations and Attrition

Two populations were defined for this report. The radiographic population comprised the 74 patients (141 feet) with complete preoperative and 12-month postoperative weight-bearing radiographs and was analyzed on a complete-case basis. Ascertainment of adverse events depended on clinical review at the treating institution, and clinical follow-up beyond the early postoperative period was documented only for children who continued follow-up at the treating institution. For the 45 eligible patients excluded from the radiographic analysis, no clinical follow-up record could be retrieved from the institutional archive. A proportion of these children are likely to have continued their follow-up in private practice, so that the corresponding records are not held by the institution and could not be accessed for this study. The safety population therefore coincides with the radiographic population, and the events reported below are adverse events recorded among the 74 patients with available 12-month radiographs and clinical follow-up rather than an estimate of the overall risk of the procedure.

To establish whether attrition had introduced selection bias, the 74 included patients were compared with the 45 eligibles but excluded patients using the variables recorded for both groups: age at surgery, sex, laterality of treatment, weight, and height. Continuous variables were compared with Welch *t* tests and categorical variables with Fisher exact tests, without adjustment for multiple comparisons. The comparison is presented as Supplementary Table S1. There was no difference in the baseline.

The reasons for incomplete imaging in this cohort are largely organizational rather than clinical. A substantial proportion of the children treated at this institution are managed privately. Their preoperative and postoperative radiographs are frequently obtained at external imaging facilities and remain in the possession of the family, and their postoperative surveillance is often continued in private practice outside the hospital. The corresponding images and clinical notes are therefore not held in the institutional archive and could not be retrieved for this analysis, irrespective of how the child had progressed. Absence from institutional radiographic follow-up in this setting may therefore largely reflect continuation of care outside the institution rather than an unfavourable outcome. This could not be verified patient by patient, and outcome-related attrition cannot be excluded.

Part of this institutional series has been reported previously in a study of return to sport after the same procedure [22]. That study was designed around a different question, applied different eligibility criteria: complete functional and sports-participation data rather than complete radiographic follow-up. The study analysed data at the level of the patient rather than the foot. The present work is therefore a separate analysis of a partial overlapping institutional cohort. Approximately 45 of the 74 patients analysed here (about 61%) were also included in that report; the overlap between the two cohorts is therefore substantial. That study reported the magnitude of angular correction only as a candidate predictor of return to sport.

**Figure 1:**
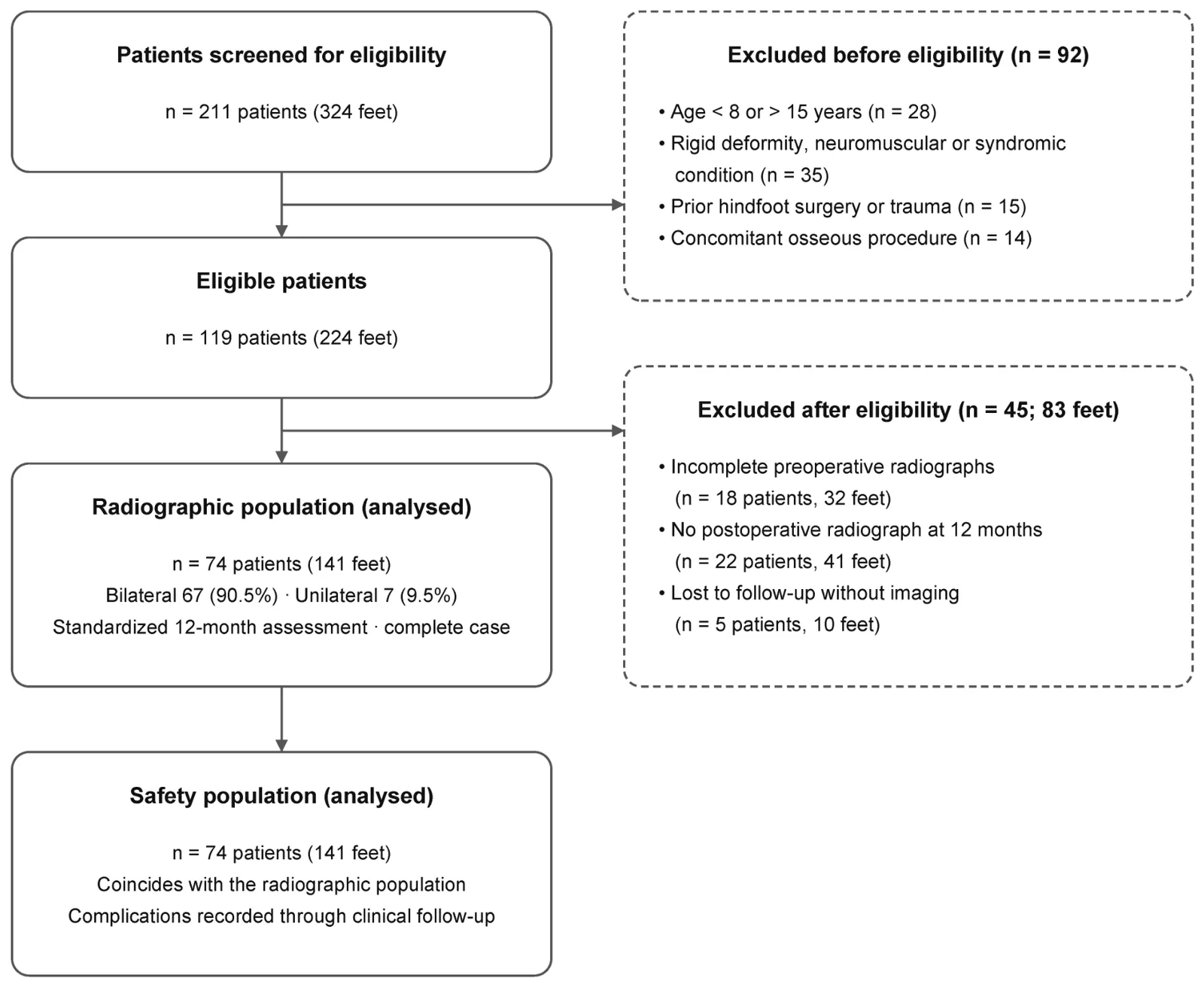
STROBE flow diagram of participant selection. The screened database comprised 211 patients and 324 distinct feet. Of these, 119 patients (224 feet) met the initial eligibility criteria. After exclusions related to radiographic completeness or follow-up, 74 patients (141 feet) were included in the 12-month radiographic analysis; complications were recorded through the available clinical follow-up.

Exclusion criteria were rigid flatfoot (including tarsal coalition or congenital vertical talus), neuromuscular or syndromic conditions, prior hindfoot surgery or trauma, concomitant osteotomy or other osseous procedure, and incomplete imaging. Of 211 screened patients, 92 were excluded before full eligibility assessment. Among 119 eligible patients (224 feet), 45 patients (83 feet) were excluded because of incomplete preoperative radiographs, absence of 12-month postoperative radiographs, or loss to follow-up without imaging. The final radiographic cohort comprised 74 patients and 141 feet, which, as detailed in Section 2.2.2, also constitutes the safety population (Figure 1).

### 2.3. Surgical Technique and Postoperative Care

Two fellowship-trained pediatric foot surgeons performed all procedures under general anesthesia with a thigh tourniquet. Through an approximately 2 cm lateral incision over the sinus tarsi, blunt dissection was performed with protection of superficial sensory branches. A blunt guidewire was advanced from lateral to medial under fluoroscopic control. Sequential trial implants were inserted, and the definitive size was chosen as the smallest implant that abolished pathological hindfoot eversion on simulated weight bearing while preserving passive subtalar inversion and a residual arc of eversion. This endpoint was assessed intraoperatively by manual stress testing of the hindfoot with the ankle in neutral dorsiflexion, and both surgeons applied the same sizing criterion. The definitive implant was a nonabsorbable titanium subtalar endorthesis (Endortesi Sub Talare—X-flat, Episcan Srl, Cesano Maderno, Italy), which was used in every foot in this series and which is manufactured in nominal sizes 9 to 14 mm. It was inserted and its position was confirmed fluoroscopically. Acceptable position was defined as full seating of the implant within the sinus tarsi and tarsal canal on the lateral view, without protrusion beyond the lateral wall of the calcaneus and without encroachment on the posterior facet.

Neither casts nor braces were used postoperatively. Weight bearing in regular footwear was permitted from postoperative day 4 after unilateral procedures and from postoperative day 7 after bilateral procedures, initially with two crutches, generally discontinued within 14 days. Low-impact activities were then resumed progressively; swimming being permitted after 1 month and other sports from 3 months according to clinical progress.

Bilateral procedures were performed during the same anesthetic session in every bilaterally treated child; no patient was operated in two stages. Standing anteroposterior and lateral radiographs were obtained routinely at 3 months postoperatively to verify implant position. The radiographs used for the outcome analysis were obtained at the standardized 12-month postoperative assessment, which forms part of the routine institutional pathway and was available for every patient included in this analysis. In children whose clinical follow-up continued beyond that point, further radiographs were sometimes obtained at later visits; these later examinations were not used for the outcome analysis. Restricting the analysis to the 12-month examination ensured that the radiographic comparison was made at an identical postoperative interval in all patients and avoided confounding of the estimated change by heterogeneous imaging intervals. The mean value of 18.3 ± 3.7 months reported in Section 3.1 describes the duration of clinical follow-up and not the timing of the radiographs entering the analysis, which was 12 months in every patient. Follow-up beyond this radiographic time point, when available, was used for clinical surveillance of pain, implant extrusion, implant removal, and reoperation. The institutional policy was implant retention: removal was not planned after a defined interval and was undertaken only in children with persistent implant-related symptoms. Accordingly, the implant was left in situ in 72 of the 74 patients (97.3%) at the time of the last follow-up; one further child, in whom the implant was retained, underwent reoperation for loss of correction, so that 71 patients (95.9%) had no further hindfoot surgery of any kind.

### 2.4. Outcomes and Measurement Reliability

The primary radiographic endpoint was change in Meary’s angle. No written statistical analysis plan was registered before data extraction; this endpoint is therefore designated as the primary radiographic endpoint rather than as a formally prespecified endpoint. Secondary radiographic endpoints were changes in the Costa–Bertani angle, Kite’s angle, and calcaneal pitch. All measurements were obtained from standing radiographs by two fellowship-trained pediatric orthopedic surgeons acting as independent assessors, who applied the anatomical landmarks and reference lines of the standard published definitions of these parameters [1,2]. Both observers measured all radiographs independently and were blinded to clinical information, including symptoms and complications, and the examinations were assessed in randomized order. No formal calibration session preceded data collection: both observers were experienced pediatric orthopedic surgeons who apply these measurements in routine clinical practice, and agreement between them was quantified as described below rather than assumed. Blinding to the time point was not achievable, because the implant is visible on postoperative radiographs; this residual source of bias is acknowledged. Interobserver reliability was evaluated for each foot using preoperative and postoperative measurements obtained independently by both observers. Intraclass correlation coefficients were calculated separately for each radiographic parameter using a two-way random-effects model with absolute agreement for single measurements, ICC(2,1). The paired observer measurements were not retained in the study database once the reliability analysis had been completed, so that confidence intervals for these coefficients could not subsequently be computed; this is acknowledged in the limitations. The value entered into the mixed-effects models was the arithmetic mean of the two independent measurements when the observers differed by 2° or less, and the consensus value obtained by joint review when they differed by more than 2°. Consensus review was performed only after the reliability analysis had been completed, so that the reported coefficients reflect fully independent measurements.

Safety outcomes were sinus tarsi pain, implant extrusion, implant removal, deep infection, neurovascular injury, and reoperation for recurrent or residual deformity. Events were identified from the clinical record for all patients in the analytic cohort, together with, where documented, the time of onset, laterality, duration, and treatment of sinus tarsi pain and the indication and timing of any implant removal or reoperation. Complications are reported both per patient and per treated foot. Age, sex, laterality, and duration of clinical follow-up were extracted from institutional records.

### 2.5. Statistical Analysis

Continuous variables are reported as mean ± standard deviation (range), and categorical variables as number (percentage). For each radiographic endpoint, a linear mixed-effects model included time (preoperative versus postoperative) as a fixed effect and random intercepts for feet nested within patients, thereby accounting for repeated measurements within each foot and correlation between feet in bilateral cases. Model residuals were inspected graphically. Estimated marginal means were used to calculate postoperative-minus-preoperative changes with 95% confidence intervals. Two-sided *p* values were obtained using Satterthwaite’s approximation. Meary’s angle was the primary radiographic endpoint; Holm adjustment was applied across the three secondary radiographic endpoints. Radiographic analysis was complete-case. Because every radiographic outcome was measured at the same standardized 12-month postoperative time point, follow-up duration was constant across the radiographic comparison. It was therefore not modelled either as a covariate or as a time-by-follow-up interaction. Variation in follow-up duration relates to clinical surveillance only and is addressed qualitatively in the limitations. Individual response was additionally summarized by classifying each foot as improved, unchanged, or worsened relative to the smallest detectable change, calculated as 1.96 × √2 × the standard error of measurement derived from the reliability analysis. This threshold identifies change exceeding measurement error and is not a validated minimal clinically important difference. The standard error of measurement was derived from the standard deviation of a single observer’s measurements; the resulting threshold is therefore deliberately conservative, and had it been derived from the mean of the two observers a larger proportion of feet would have been classified as improved. Analyses were performed using R version 4.4.2 with the lme4, lmerTest, and emmeans packages. A *p* value < 0.05 was considered statistically significant.

## 3. Results

### 3.1. Participant Selection and Characteristics

The final cohort included 74 patients and 141 feet. Forty-seven patients (63.5%) were male and 27 (36.5%) were female. Mean age at surgery was 11.5 ± 1.6 years (range, 9–15 years). Sixty-seven patients (90.5%) underwent bilateral procedures and seven (9.5%) underwent unilateral procedures. Radiographic outcomes were assessed at 12 months in all patients. Mean clinical follow-up was 18.3 ± 3.7 months (median, 18.7 months; interquartile range, 15.2–20.9 months; range, 10.1–24.5 months) (Table 1).

The 74 included patients and the 45 eligible patients excluded because of incomplete imaging did not differ significantly in age at surgery (11.5 ± 1.6 versus 11.9 ± 1.7 years; *p* = 0.21), sex distribution (63.5% versus 71.1% male; *p* = 0.43), laterality of treatment (90.5% versus 84.4% bilateral; *p* = 0.38), weight (*p* = 0.30), or height (*p* = 0.42) (Supplementary Table S1). These variables are, however, the only ones recorded for both groups; because the reason for non-attendance could not be established for individual patients, differences in symptom burden, implant tolerance, or satisfaction between the two groups cannot be excluded.

### 3.2. Radiographic Outcomes

**Figure 2:**
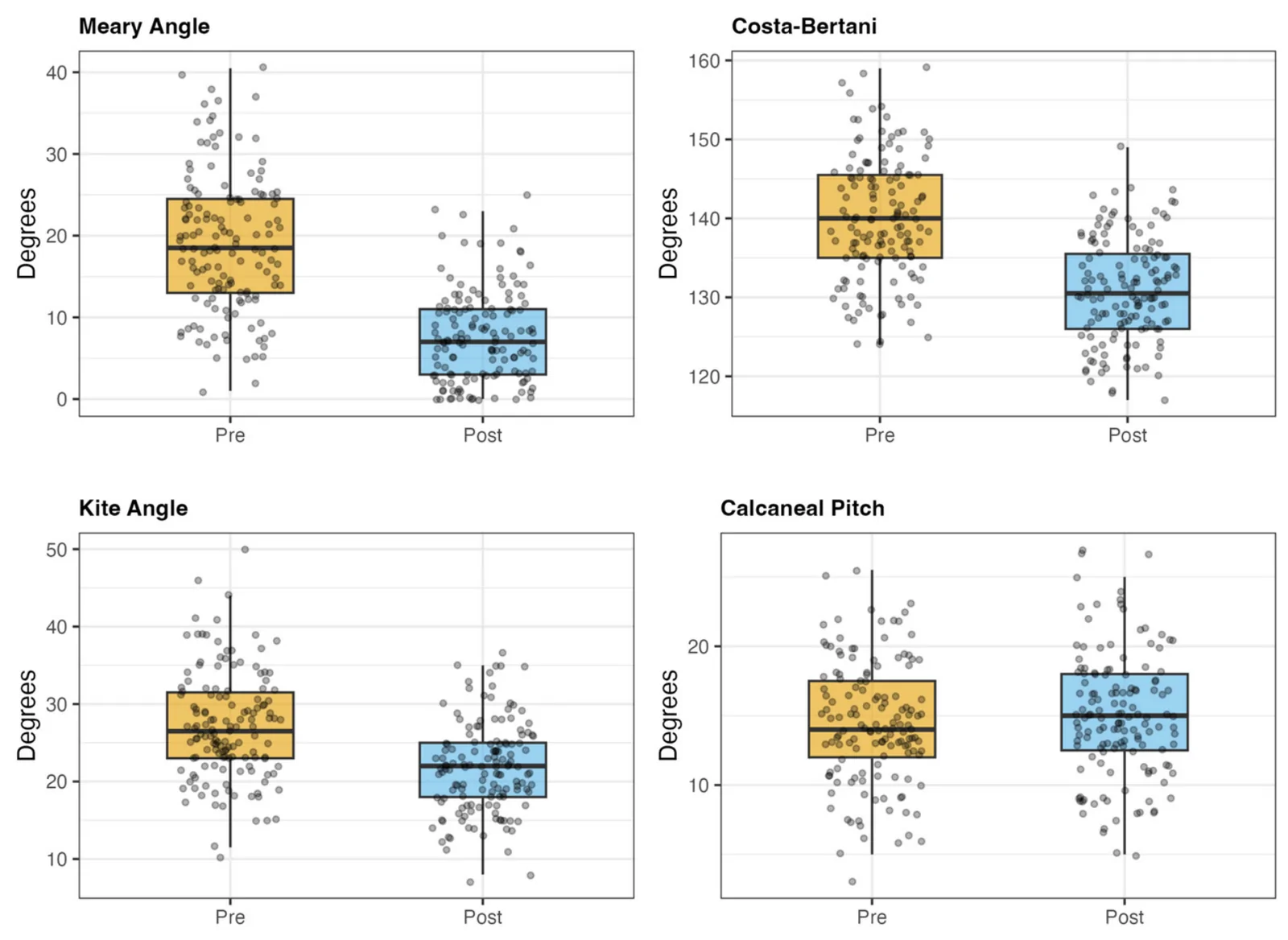
Preoperative and postoperative radiographic measurements in the complete-case cohort (*n* = 141 feet at each time point). Boxes show the interquartile range, central lines the median, whiskers 1.5 times the interquartile range, and points individual feet. Inferential estimates account for clustering (Table 2); group-level improvement does not imply normalization in every foot.

Interobserver reliability was good to excellent for all measurements. ICC(2,1) values were 0.89 for Meary’s angle, 0.91 for the Costa–Bertani angle, 0.85 for Kite’s angle, and 0.90 for calcaneal pitch, based on independent preoperative and postoperative measurements by both observers; confidence intervals could not be computed because the paired observer measurements were not retained. All four radiographic parameters improved after surgery (Table 2; Figure 2). Meary’s angle decreased from 19.0° ± 8.5° to 7.7° ± 5.6° (model-based mean change, −11.3°; 95% CI, −12.8 to −9.8; *p* < 0.001). The Costa–Bertani angle decreased by 9.0° (95% CI, −10.1 to −7.9) and Kite’s angle by 5.5° (95% CI, −6.6 to −4.4), while calcaneal pitch increased by 1.7° (95% CI, 1.0 to 2.4). Holm-adjusted *p* values are reported in Table 2. The increase in calcaneal pitch, although formally significant, was small in magnitude and did not exceed the smallest detectable change for this parameter (3.8°); its clinical relevance therefore remains uncertain, particularly in the absence of a validated clinical threshold for this measurement.

Individual responses were more variable than the group means indicate. The smallest detectable change was 7.8° for Meary’s angle, 6.5° for the Costa–Bertani angle, 7.5° for Kite’s angle, and 3.8° for calcaneal pitch. Using these thresholds, 94 of 141 feet (66.7%) improved, 47 (33.3%) were unchanged and none worsened for Meary’s angle; 99 feet (70.2%) improved, 42 (29.8%) were unchanged and none worsened for the Costa–Bertani angle; 43 feet (30.5%) improved, 96 (68.1%) were unchanged and 2 (1.4%) worsened for Kite’s angle; and 16 feet (11.3%) improved, 115 (81.6%) were unchanged and 10 (7.1%) worsened for calcaneal pitch. Group-level improvement therefore did not correspond to a change exceeding measurement error in every foot, and this was most evident for calcaneal pitch. Individual preoperative-to-postoperative trajectories are shown in Supplementary Figure S1.

### 3.3. Safety Outcomes

Sinus tarsi pain was recorded in 13 patients (17.6%), affecting 23 of the 141 treated feet (16.3%); the pain was bilateral in 10 patients and unilateral in 3. Symptoms began in the immediate postoperative period in all affected children and were persistent rather than intermittent. Treatment consisted of physiotherapy and oral non-steroidal anti-inflammatory drugs. Pain resolved with nonoperative treatment in 11 of these 13 patients, after approximately 2 months. One patient (1.4%) experienced implant extrusion. Two patients (2.7%) underwent implant removal for persistent symptoms, and one patient (1.4%) underwent reoperation for loss of correction. No deep infection or neurovascular injury was recorded (Table 3).

The complication categories overlapped as follows. Both patients who underwent implant removal were among the 13 patients with sinus tarsi pain. These were the two children in whom the pain did not resolve, and in each of them the implant was removed from one foot after at least 6 months of nonoperative treatment. One of these two children was also the patient recorded as having implant extrusion. Therefore, that extrusion and removal refer to the same child rather than to two independent events. The patient who underwent reoperation for loss of correction had been treated bilaterally and was reoperated on both feet. This child had also reported discomfort, although it had not been recorded as sinus tarsi pain requiring treatment. At the level of the treated foot, sinus tarsi pain affected 23 of the 141 feet, implant extrusion 1 of 141, implant removal 2 of 141, and reoperation for loss of correction 2 of 141. The implant was therefore not removed in 72 of the 74 patients (97.3%), and 71 patients (95.9%) underwent no further hindfoot surgery of any kind by the time of the last follow-up.

## 4. Discussion

In this retrospective cohort of 74 children and 141 feet, isolated nonabsorbable endosinotarsal screw arthroereisis was associated with improvement in all four radiographic measures at the standardized 12-month radiographic assessment. Change was greatest for Meary’s and Costa–Bertani angles, while calcaneal pitch improved to a smaller extent. These findings describe a reproducible radiographic response over the observed period. Major recorded adverse events and reoperations were uncommon among the patients with available follow-up, whereas sinus tarsi pain was recorded in approximately one in six patients; this description should not be read as evidence that rare or late complications have been excluded. The design does not permit conclusions about superiority over nonoperative care or alternative operations and, in the absence of a control group, the observed radiographic changes cannot be attributed exclusively to the implant, since physiological growth and maturation of the pediatric foot may have contributed during the follow-up interval.

The magnitude and direction of radiographic change are broadly consistent with previous pediatric arthroereisis series and systematic reviews [8,9,10,11,12,13,14,15,16,17,19,23,24,25,26]. De Pellegrin et al. reported substantial correction with extra-articular screw arthroereisis, while studies of other implant designs likewise found improvements in talar–first metatarsal and talocalcaneal alignment [8,12,13,15,16,19,24,25,26]. Cross-study numerical comparison remains imprecise because cohorts differ in baseline severity, age, implant design, concomitant soft-tissue procedures, follow-up duration, and radiographic definitions [9,10,11,14]. The present study adds a relatively large foot-level dataset for an isolated screw technique and explicitly accounts for the high proportion of bilateral cases.

The pattern of change is biomechanically plausible. Arthroereisis restrains excessive subtalar eversion and limits pathological talar–calcaneal motion without performing an osteotomy. The larger changes in measures reflecting talar alignment and medial arch collapse, with a smaller increase in calcaneal pitch, are consistent with this mechanism. Gait-analysis data after pediatric arthroereisis have demonstrated improved frontal-plane rearfoot orientation, reduced pathological pronation–supination at the ankle and midtarsal joints, and more physiological tibialis anterior activation [20]. These dynamic changes provide a credible mechanism by which reducing excessive pronation, without necessarily abolishing it, may improve loading and muscular control.

Importantly, the absolute postoperative values in our cohort indicate partial correction rather than uniform normalization. The mean postoperative Costa–Bertani angle was 130.9°, near the upper boundary of commonly used reference ranges, while mean postoperative Meary’s angle (7.7°), Kite’s angle (21.7°), and calcaneal pitch (17.2°) likewise suggest residual radiographic deformity in a proportion of feet [1,2,27,28]. Published reference values vary with age, population, radiographic technique, and the definition adopted for each angle, and a recent case-control study found that only three of nine commonly used radiographic parameters yielded values that reliably distinguished normal feet from flatfeet [28]. A dichotomous normal-abnormal classification is therefore avoided here in favour of the concept of partial correction. This does not necessarily represent treatment failure. Flexible flatfoot exists along a continuum, and the presence or intensity of symptoms is not determined solely by static valgus or arch height. In the comparative radiographic study by Moraleda and Mubarak, symptomatic and asymptomatic flexible flatfeet differed in only two of 14 measurements; conventional measures of hindfoot alignment and the longitudinal arch did not consistently distinguish symptomatic status [5]. Thus, the clinical objective is not simply to force every angle into a nominal reference interval, but to reduce pathological mechanics sufficiently to relieve symptoms and improve function while preserving subtalar mobility.

The converse is equally important: radiographic improvement alone cannot establish clinical success. Studies incorporating clinical instruments have reported concurrent improvements in pain, AOFAS, OxAFQ-C, quality of life, or gait after arthroereisis [19,20,26,29,30], and long-term data suggest that selected postoperative alignment measures may correlate with functional outcomes [31]. Nevertheless, these associations do not imply that the magnitude of angular change is the principal determinant of recovery. In our previously published institutional return-to-sport cohort, neither ΔMeary nor residual pain independently predicted return to sport, whereas early postoperative activity level was the strongest correlate [22]. Taken together, the literature supports a two-domain interpretation: radiographs document the mechanical response to treatment, whereas symptoms, activity, participation, and patient-reported function determine clinical success.

The present dataset was designed to address the radiographic domain and the complications recorded in the clinical notes. It cannot determine whether greater angular improvement produced less pain, better footwear tolerance, higher participation, or better quality of life because standardized preoperative and postoperative patient-reported outcomes were not collected. Our findings should therefore not be framed as proof of clinical effectiveness. Rather, they show a reproducible reduction in the structural components of planovalgus deformity, achieved without requiring complete radiographic normalization and with a low rate of major reintervention. This distinction is clinically relevant when counseling families: residual flatfoot morphology may persist despite a potentially useful reduction in pronatory mechanics, and follow-up should prioritize symptoms and function rather than radiographs alone.

Sinus tarsi pain was the most frequent adverse event, consistent with the established complication profile of arthroereisis [7,9,10,13,15,16,24]. Most cases resolved without further surgery, but the 17.6% incidence is clinically relevant and should be included in preoperative counseling. Persistent pain warrants evaluation of implant size and position, overconstraint, local irritation, and alternative pain generators. The rates of implant removal, extrusion, and reoperation were low in this cohort, but the confidence with which rare harms can be excluded is limited by sample size and follow-up duration. Implant position may influence sinus tarsi pain, extrusion, and loss of correction, and we considered assessing it retrospectively. Published measurements of implant position require weight-bearing anteroposterior and lateral views acquired for that purpose, from which implant orientation relative to the talar axis, medial depth, and distance from the lateral calcaneal wall are derived [32]; computed tomography localises the implant more precisely but is not part of routine pediatric follow-up [33]. Our radiographs were obtained to assess hindfoot alignment, without a calibration marker or standardised positioning, and no dedicated implant-position view is acquired at our institution. No reproducible measurement could therefore be derived, and we report this rather than offer an unvalidated estimate in a handful of patients. The lack of a validated implant-position measure limits this cohort and the arthroereisis literature generally; prospective studies should acquire dedicated views at the time of postoperative imaging.

Whether implant material modifies long-term outcome remains unresolved. Bioabsorbable implants have shown satisfactory correction in selected cohorts, including follow-up beyond 4 years, whereas nonabsorbable devices offer persistent mechanical restraint but may require removal for intolerance [11,18,23]. Existing comparative evidence is heterogeneous and does not justify claiming that one material is uniformly safer or more durable [10]. Our results support the performance of the studied nonabsorbable screw over the observed interval; they do not establish superiority over absorbable implants.

A methodological strength is the use of mixed-effects models with feet nested within patients. This preserves foot-level information while avoiding the false precision produced by treating bilateral feet as independent. Independent measurements by two observers demonstrated good to excellent agreement across all four parameters (ICC range, 0.85–0.91), supporting the reproducibility of the radiographic endpoints. Consecutive case identification, an explicit flow diagram, isolated arthroereisis without concomitant osseous procedures, and a standardized 12-month radiographic assessment also improve interpretability.

## 5. Limitations

The retrospective, single-center design is open to selection bias, unmeasured confounding, and incomplete documentation. Attrition is the chief concern: 45 of 119 eligible patients (37.8%) lacked the radiographs required for analysis. Children with persistent symptoms, implant intolerance, treatment elsewhere, or dissatisfaction may be more or less likely to return for imaging, and the direction of any resulting bias cannot be determined from our data. Two considerations temper this. Non-attendance may largely reflect care continued privately outside the institution rather than a poor outcome (Section 2.2.2), although this could not be confirmed for individual patients and outcome-related attrition cannot be excluded, and included and excluded patients did not differ in age, sex, laterality, weight, or height (Supplementary Table S1). Both arguments rest on routinely recorded variables and cannot exclude differences in symptom burden or implant tolerance. Because complications were ascertained through the same follow-up, the reported frequencies describe the analyzed cohort and may under- or overestimate those in the full operated population.

Without a nonoperative or surgical comparison group, causality and comparative effectiveness cannot be inferred. Body mass index, ligamentous laxity, gastrocnemius–soleus tightness, baseline activity, adherence to rehabilitation, implant position, and implant size were unavailable for adjusted analysis, and the paired observer measurements were not retained, so confidence intervals for the intraclass correlation coefficients cannot be reported. We therefore attempted no analysis of predictors: with 13 children affected by sinus tarsi pain and single instances of extrusion and reoperation, the events are too few for multivariable modelling. Radiographic outcomes were measured at the same 12-month time point in every patient, so variable clinical follow-up did not affect the timing of the radiographic comparison, although it may have affected ascertainment of later pain, extrusion, removal, or reoperation. Patient-reported outcomes, functional testing, and standardized pain trajectories were not collected, and a mean clinical follow-up of 18.3 months cannot establish maintenance of correction to skeletal maturity, late implant intolerance, degenerative change, or recurrence. The findings apply to one implant in the hands of experienced surgeons.

Prospective multicenter studies with standardized indications, implant-position measures, and predefined confounders are needed, assessing imaging alongside patient-reported pain and function, participation and return to sport, and gait or plantar-pressure data. Comparative work should set nonabsorbable against absorbable implants, and arthroereisis against optimized nonoperative treatment or established reconstructive procedures, with follow-up to skeletal maturity to establish durability, implant survivorship, and late joint health. That the implant was not removed in 97.3% of these children also bears on the current debate about arthroereisis in adults with symptomatic flexible flatfoot, where evidence remains scarce; whether a device intended for indefinite retention behaves the same way in the mature foot cannot be inferred from pediatric data and needs dedicated study.

## 6. Conclusions

Nonabsorbable endosinotarsal screw arthroereisis was associated with improvement in Meary’s, Costa–Bertani, and Kite’s angles and a smaller increase in calcaneal pitch at the standardized 12-month radiographic assessment in selected children with symptomatic flexible flatfoot. The postoperative mean values indicate partial correction, with residual radiographic flatfoot likely persisting in a proportion of feet. Major recorded adverse events and reoperations were uncommon among the patients with available follow-up, whereas sinus tarsi pain was recorded in 17.6% of patients, and the implant was not removed in 97.3% of patients, with 95.9% undergoing no further hindfoot surgery of any kind. These observations describe what was recorded in this cohort and do not exclude rare or late complications. Because standardized symptom-based and functional outcomes were unavailable and no control group was studied, clinical benefit cannot be established and the radiographic changes cannot be attributed exclusively to the procedure. Prospective studies integrating radiographic, patient-reported, and functional outcomes are required.

## Figures and Tables

**Table 1:** Demographic and clinical characteristics.

Characteristic	Value
Patients, *n*	74
Feet, *n*	141
Age at surgery, years	11.5 ± 1.6 (9–15)
Male sex	47 (63.5%)
Female sex	27 (36.5%)
Bilateral procedures	67 (90.5%)
Unilateral procedures	7 (9.5%)
Clinical follow-up, months	18.3 ± 3.7

Data are mean ± standard deviation (range) or *n* (%), unless otherwise indicated.

**Table 2:** Preoperative and postoperative radiographic outcomes.

Parameter	Preoperative, Mean ± SD	Postoperative, Mean ± SD	Mean Change (95% CI)	Adjusted *p* Value
Meary’s angle (°)	19.0 ± 8.5	7.7 ± 5.6	−11.3 (−12.8 to −9.8)	<0.001 *
Costa–Bertani angle (°)	139.9 ± 7.8	130.9 ± 6.5	−9.0 (−10.1 to −7.9)	<0.001
Kite’s angle (°)	27.2 ± 7.0	21.7 ± 5.8	−5.5 (−6.6 to −4.4)	<0.001
Calcaneal pitch (°)	14.5 ± 4.4	16.2 ± 4.3	+1.7 (1.0 to 2.4)	0.001

CI, confidence interval; SD, standard deviation. Changes are postoperative minus preoperative estimates from linear mixed-effects models. * Unadjusted *p* value for the primary radiographic endpoint; Holm-adjusted *p* values are shown for secondary endpoints.

**Table 3:** Complications recorded in the analyzed cohort (*n* = 74 patients, 141 feet).

Outcome	Patients, *n* (%)
Sinus tarsi pain	13 (17.6)
Resolved nonoperatively among pain cases	11/13 (84.6)
Implant extrusion	1 (1.4)
Implant removal	2 (2.7)
Reoperation for loss of correction	1 (1.4)
Deep infection	0 (0)
Neurovascular injury	0 (0)

Percentages use the 74-patient cohort as denominator unless otherwise specified. Categories may overlap; the observed overlap between categories and the corresponding foot-level counts are described in Section 3.3.

## Data Availability

The datasets generated and/or analyzed during the current study are not publicly available because of institutional restrictions on the sharing of clinical data but are available from the corresponding author upon reasonable request and subject to applicable privacy and institutional requirements.

## Supplementary Materials

The following supporting information can be downloaded at: https://www.mdpi.com/article/10.3390/jcm15207950/s1. Table S1, comparison of the 74 included patients and the 45 eligible but excluded patients; Figure S1, individual preoperative-to-postoperative trajectories of all 141 feet for each radiographic parameter, classified as improved, unchanged, or worsened against the smallest detectable change; and STROBE Statement—Checklist of items that should be included in reports of cohort studies.
