# Supplementary material for: Radiographic Changes and Recorded Complications After Nonabsorbable Endosinotarsal Screw Arthroereisis for Symptomatic Pediatric Flexible Flatfoot: A Retrospective Cohort Study
Source: J Clin Med. 2026 Oct 14;15(20):7950. doi: 10.3390/jcm15207950 (PMC13706615; doi:10.3390/jcm15207950)
Supplement: Supplementary file 1 [file jcm-15-07950-s001.zip › jcm-4464030-supplementary.pdf]

Supplementary Materials

Manuscript ID: jcm-4464030 · Radiographic Changes and Recorded Complications After Nonabsorbable Endosinotarsal Screw Arthroereisis for Symptomatic Pediatric Flexible Flatfoot: A Retrospective Cohort Study

Table S1. Comparison of the 74 included patients and the 45 eligible but excluded patients.

Variable	Included (n = 74)	Excluded (n = 45)	p value	Test
Age at surgery, years	11.5 ± 1.6	11.9 ± 1.7	0.21	Welch t test
Male sex, n (%)	47 (63.5)	32 (71.1)	0.43	Fisher exact test
Bilateral treatment, n (%)	67 (90.5)	38 (84.4)	0.38	Fisher exact test
Weight, kg	55.3 ± 2.1	52.5 ± 1.8	0.30	Welch t test
Height, cm	153.4 ± 5.3	156.2 ± 6.2	0.42	Welch t test

Data are mean ± standard deviation or n (%). Continuous variables were compared with Welch t tests and categorical variables with Fisher exact tests, without adjustment for multiple comparisons. These are the only variables recorded for both groups; the comparison cannot exclude differences in symptom burden, implant tolerance, or satisfaction.

Figure S1. Individual preoperative-to-postoperative trajectories of all 141 feet for each radiographic parameter, classified as improved, unchanged, or worsened against the smallest detectable change.

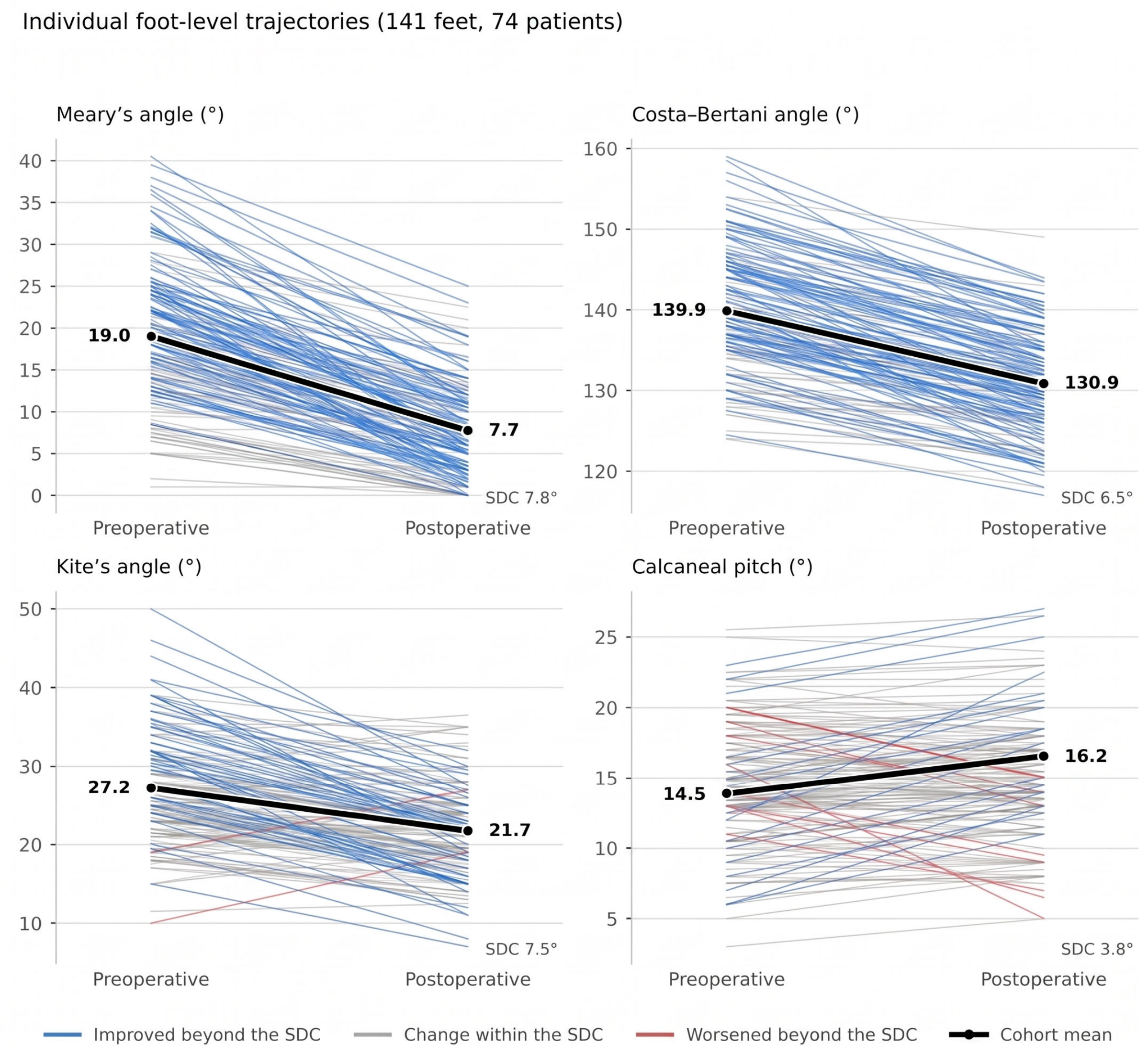

STROBE Statement — Checklist of items that should be included in reports of cohort studies

Manuscript ID: jcm-4464030 · **Title:** Radiographic Changes and Recorded Complications After Nonabsorbable Endosinotarsal Screw Arthroereisis for Symptomatic Pediatric Flexible Flatfoot: A Retrospective Cohort Study

Section / Topic	Item	Recommendation	Reported in
Title and abstract	1	(a) Indicate the study's design with a commonly used term in the title or the abstract	Title; Abstract ("A Retrospective Cohort Study")
		(b) Provide in the abstract an informative and balanced summary of what was done and what was found	Abstract, Background/Objectives through Conclusions
**Introduction**			
Background/rationale	2	Explain the scientific background and rationale for the investigation being reported	Introduction, paragraphs 1–4
Objectives	3	State specific objectives, including any prespecified hypotheses	Introduction, final paragraph
**Methods**			
Study design	4	Present key elements of study design early in the paper	Section 2.1
Setting	5	Describe the setting, locations, and relevant dates, including periods of recruitment, exposure, follow-up, and data collection	Section 2.1; Section 2.3; Section 3.1
Participants	6	(a) Give the eligibility criteria, and the sources and methods of selection of participants. Describe methods of follow-up	Sections 2.2, 2.2.1, 2.2.2; Figure 1
		(b) For matched studies, give matching criteria and number of exposed and unexposed	Not applicable — no matching was performed
Variables	7	Clearly define all outcomes, exposures, predictors, potential confounders, and effect modifiers. Give diagnostic criteria, if applicable	Sections 2.2.1, 2.4
Data sources/measurement	8	For each variable of interest, give sources of data and details of methods of assessment. Describe comparability of assessment methods if there is more than one group	Section 2.4 (measurement protocol, blinding, reliability); Section 2.2.2
Bias	9	Describe any efforts to address potential sources of bias	Section 2.2.2 (populations and attrition); Section 2.4 (independent assessors, blinding, randomized order, consensus rule); Discussion, limitations
Study size	10	Explain how the study size was arrived at	Section 2.2 (consecutive series, all eligible patients); Figure 1
Quantitative variables	11	Explain how quantitative variables were handled in the analyses. If applicable, describe which groupings were chosen and why	Section 2.5 (continuous angles; responder classification against the smallest detectable change)
Statistical methods	12	(a) Describe all statistical methods, including those used to control for confounding	Section 2.5 (linear mixed-effects models, feet nested within patients)
		(b) Describe any methods used to examine subgroups and interactions	Not applicable — radiographic outcomes were assessed at a single standardized 12-month time point, and no subgroup or interaction analysis was performed
		(c) Explain how missing data were addressed	Section 2.2.2; Section 2.5 (complete-case analysis, no imputation)
		(d) If applicable, explain how loss to follow-up was addressed	Section 2.2.2; Section 3.1; Discussion, limitations
		(e) Describe any sensitivity analyses	Not applicable — because all radiographic outcomes were measured at the same 12-month time point, no sensitivity analysis on follow-up duration was required (Section 2.5)
**Results**			
Participants	13	(a) Report numbers of individuals at each stage of the study	Section 2.2; Section 3.1; Figure 1
		(b) Give reasons for non-participation at each stage	Section 2.2; Figure 1
		(c) Consider use of a flow diagram	Figure 1
Descriptive data	14	(a) Give characteristics of study participants and information on exposures and potential confounders	Section 3.1; Table 1
		(b) Indicate number of participants with missing data for each variable of interest	Section 2.2.2; Section 3.1; Figure 1
		(c) Summarise follow-up time	Section 3.1; Table 1 (standardized 12-month radiographic assessment; clinical follow-up

			18.3 ± 3.7 months)
Outcome data	15	Report numbers of outcome events or summary measures over time	Section 3.2 (Table 2, Figure 2, Supplementary Figure S1); Section 3.3 (Table 3)
Main results	16	(a) Give unadjusted estimates and, if applicable, confounder-adjusted estimates and their precision. Make clear which confounders were adjusted for and why they were included	Section 3.2; Table 2 (model-based mean changes with 95% CI; Holm adjustment for secondary endpoints)
		(b) Report category boundaries when continuous variables were categorized	Section 2.5; Section 3.2 (smallest detectable change thresholds)
		(c) If relevant, consider translating estimates of relative risk into absolute risk for a meaningful time period	Section 3.3 (patient-level and foot-level event counts)
Other analyses	17	Report other analyses done — e.g. analyses of subgroups and interactions, and sensitivity analyses	Section 3.2 (individual-response analysis against the smallest detectable change; Supplementary Figure S1)
**Discussion**			
Key results	18	Summarise key results with reference to study objectives	Discussion, first paragraph; Conclusions
Limitations	19	Discuss limitations of the study, taking into account sources of potential bias or imprecision. Discuss both direction and magnitude of any potential bias	Discussion, limitations paragraph (attrition as the principal limitation; unavailable comparison of included and excluded patients; absent implant-position and implant-size data; no ICC confidence intervals; no control group)
Interpretation	20	Give a cautious overall interpretation of results considering objectives, limitations, multiplicity of analyses, results from similar studies, and other relevant evidence	Discussion, paragraphs on partial correction, the two-domain interpretation and causal caution; Conclusions
Generalisability	21	Discuss the generalisability (external validity) of the study results	Discussion, limitations (single centre, one device, experienced surgeons)
**Other information**			
Funding	22	Give the source of funding and the role of the funders for the present study and, if applicable, for the original study on which the present article is based	Funding statement

Note. An Explanation and Elaboration article discusses each checklist item and gives methodological background and published examples of transparent reporting. The STROBE checklist is best used in conjunction with this article (freely available on the websites of PLoS Medicine at http://www.plosmedicine.org/, Annals of Internal Medicine at http://www.annals.org/, and Epidemiology at http://www.epidem.com/). Information on the STROBE Initiative is available at www.strobe-statement.org.
